# The Capability of Tyramine Production and Correlation between Phenotypic and Genetic Characteristics of *Enterococcus faecium* and *Enterococcus faecalis* Strains

**DOI:** 10.3389/fmicb.2015.01371

**Published:** 2015-12-08

**Authors:** Eleonora Bargossi, Fausto Gardini, Veronica Gatto, Chiara Montanari, Sandra Torriani, Giulia Tabanelli

**Affiliations:** ^1^Department of Agricultural and Food Sciences, University of BolognaCesena, Italy; ^2^Interdepartmental Center for Industrial Agri-Food Research, University of BolognaCesena, Italy; ^3^Department of Biotechnology, University of VeronaVerona, Italy

**Keywords:** *Enterococcus faecium*, *Enterococcus faecalis*, tyramine, tyrosine decarboxylase activity, gene expression

## Abstract

The aim of this study was to investigate the diversity of tyramine production capability of four *Enterococcus* strains in buffered systems in relation to their genetic characteristics and environmental conditions. Cells of the strains *Enterococcus faecalis* EF37 and ATCC 29212, and *E. faecium* FC12 and FC643 were re-suspended in phosphate/citrate buffers with different pH, NaCl concentration and incubation temperature. At intervals, cell viability and tyramine production were assessed by plate counting and HPLC analysis, respectively. The activity of a purified tyrosine decarboxylase (TDC) was determined under the same conditions, as a reference. Reduced loss in cell viability was observed in all the tested conditions, except for pH 4 after 24 h. The TDC activity was greatly heterogeneous within the enterococci: EF37 and FC12 produced the higher tyramine concentrations, ATCC 29212 showed a reduced decarboxylase activity, while EF643 did not accumulate detectable amounts of tyramine in all the conditions assayed. Among the considerate variables, temperature was the most influencing factor on tyramine accumulation for enterococcal cells. To further correlate the phenotypic and genetic characteristics of the enterococci, the TDC operon region carrying the genes tyrosine decarboxylase (*tyrDC*), tyrosine/tyramine permease (*tyrP*), and Na^+^/H^+^ antiporter (*nhaC-2*) was amplified and sequenced. The genetic organization and nucleotide sequence of this operon region were highly conserved in the enterococcal strains of the same species. The heterogeneity in tyramine production found between the two *E. faecalis* strains could be ascribed to different regulation mechanisms not yet elucidated. On the contrary, a codon stop was identified in the translated *tyrDC* sequence of *E. faecium* FC643, supporting its inability to accumulate tyramine in the tested conditions. In addition, the presence of an additional putative tyrosine decarboxylase with different substrate specificity and genetic organization was noticed for the first time. Concluding, the high TDC activity heterogeneity within enterococci determined different accumulation of tyramine, depending on different genetic determinants, regulation mechanisms, and environmental factors. The present research contributes to elucidate the genetic characteristics of enterococcal strains and correlate specific mutations to their different strain-dependent tyraminogenic activity.

## Introduction

Tyramine is a biogenic amine (BA) which can have severe acute effects if ingested in excessive amounts with food consisting in peripheral vasoconstriction, increased cardiac output, accelerated respiration, elevated blood glucose and release of norepinephrine, symptoms known also as “cheese reaction” (Shalaby, 1994; McCabe-Sellers et al., 2006; Marcobal et al., 2012).

Lactic acid bacteria (LAB) are among the most efficient producers of tyrosine decarboxylase (TDC), the enzyme responsible for tyramine formation (Marcobal et al., 2012). Among LAB, species belonging to the genus *Enterococcus* are recognized as the most efficient tyramine producers (Leuschner et al., 1999; Suzzi and Gardini, 2003; Ladero et al., 2012; Marcobal et al., 2012). BA formation provides metabolic energy and/or resistance against acidic stress (Molenaar et al., 1993; Fernández and Zúñiga, 2006; Pereira et al., 2009).

Enterococci occur in many different habitats and they are often contaminant in food of animal origin (Franz et al., 2011). Due to their salt and pH tolerance and to their ability to grow over a wide temperature range, these LAB are particularly competitive in harsh environmental conditions, and can be a relevant component of the ripening microbiota of cheeses and sausages (Franz et al., 1999, 2011; Giraffa, 2003). In addition, some strains showed probiotic features, and produce bacteriocins able to limit the growth of pathogenic and degradative microorganisms (Beshkova and Frengova, 2012; Fontana et al., 2015). On the other hand, enterococci are among the most common nosocomial opportunistic pathogens because of their antibiotic resistance often carried on mobile genetic elements transferable to other microorganisms (Giraffa, 2002; Klein, 2003; Rossi et al., 2014). Moreover, several enterococcal virulence factors have been described, such as cytolysins, aggregation substances, gelatinase extracellular surface proteins (Foulquié Moreno et al., 2006; Hollenbeck and Rice, 2012). A further matter of concern with respect to the safety of enterococci is their tyraminogenic capacity (Suzzi and Gardini, 2003; Foulquié Moreno et al., 2006; Komprda et al., 2008; EFSA, 2011). In fact, the ability to produce tyramine is considered a species characteristic of *Enterococcus faecalis* and it is extremely widespread among strains of *E. faecium* and *E. durans* (Ladero et al., 2012).

Tyrosine decarboxylase is a membrane located enzyme with large hydrophobic regions, which can efficiently work in a wide range of conditions also outside of the cells, as demonstrated in *Lactobacillus brevis* (Moreno-Arribas and Lonvaud-Funel, 2001) and in *E. faecium* and *E. faecalis* (Liu et al., 2014). In any case, tyramine is often accumulated by enterococci in higher amount already during the late exponential growth, before stationary phase, suggesting that this decarboxylation activity is not necessarily a response to starvation or nutrient depletion, and no competition between sugar catabolism and amino acid decarboxylation was observed (Pessione et al., 2009; Bargossi et al., 2015). The tyramine formed inside microbial cells through the action of TDC, is successively excreted in the environment by the cells in exchange with tyrosine through the action of the antiporter tyrosine/tyramine permease (Marcobal et al., 2012).

The proteins involved in the tyramine pathway are encoded by the TDC gene cluster, which has been described in detail in various enterococcal species, such as *E. faecalis* JH2-2 (Connil et al., 2002), *E. faecium* RM58 (Marcobal et al., 2006), and *E. durans* IPLA 655 (Ladero et al., 2013), and it has also been annotated in the genome sequence of other enterococci. All the tyramine biosynthetic loci revealed a high similarity either in gene sequence and organization (Marcobal et al., 2012). This locus usually contains the genes encoding a tyrosyl tRNA synthetase (*tyrS*), the tyrosine decarboxylase (*tyrDC*), a tyrosine/tyramine permease (*tyrP*), and a Na+/H+ antiporter (*nhaC-2*; Linares et al., 2011). However, reverse transcription-PCR analyses demonstrated that different strains can have different transcriptional organizations of the TDC gene cluster and *tyrS* is often transcribed independently and not included in the catabolic operon (Perez et al., 2015).

The relationships between the presence of enterococci and the accumulation of tyramine has been demonstrated in several fermented food, such as fermented sausages (Gardini et al., 2008), cheeses (Linares et al., 2011), and wine (Pérez-Martín et al., 2014). However, not all the strains able to decarboxylate tyrosine were characterized by the same phenotypic potential in relation to the kinetics of tyramine accumulation (Bargossi et al., 2015).

While the mechanisms of action and the role of TDC in LAB are well elucidated (Wolken et al., 2006; Pereira et al., 2009; Pessione et al., 2009), the effects on the potential decarboxylase activity of enterococcal cells in relation the main environmental factors need to be further investigated. The production of tyramine observed during the growth in laboratory media of tyraminogenic *E. faecalis* and *E. faecium* strains has been modeled in relation to environmental factors such as NaCl and tyrosine concentration, pH, pyridoxal-5-phospate supplementation and temperature (Gardini et al., 2001, 2008; Marcobal et al., 2006). The effects of carbon source, tyrosine and tyramine concentration, and pH on tyramine accumulation during the growth of *E. durans* were described by Fernández et al. (2007). In addition, Liu et al. (2014) characterized the TDC activity of two strains of *E. faecalis* and *E. faecium*, heterologously expressed in *Escherichia coli* and purified in relation to temperature, NaCl concentration and pH.

The aim of this paper was to investigate the diversity of tyramine production capability of four *Enterococcus* strains in buffered systems in relation to their genetic characteristics and environmental conditions. The strains *E. faecalis* EF37 and ATCC 29212, and *E. faecium* FC12 and FC643 were chosen for their different behavior in tyramine accumulation during the growth in culture media (Bargossi et al., 2015). In detail, we evaluated the functionality of the TDC pathway in stationary phase cells re-suspended in buffers with different pH, NaCl concentration or incubation temperature. The activity of a purified TDC was also assessed under the same conditions, as a reference. Finally, the nucleotide sequence of the TDC operon region carrying the genes *tyrDC, tyrP*, and *nhaC-2* was determined.

## Materials and Methods

### Enterococcal Strains and Evaluation of TDC Activity in Phosphate/Citrate Buffer

The strains *E. faecalis* EF37 and ATCC 29212, *E. faecium* FC12 and FC643 were stored in 20% (w/v) glycerol at -80°C and pre-cultivated twice for 24 h at 37°C in BHI Broth (Oxoid, Basingstoke, UK) added with 4.4 mM tyrosine (Sigma–Aldrich, Gallarate, Italy).

After 24 h of pre-cultivation, the cells were collected by centrifugation at 8000 × *g* for 10 min and washed twice with physiological solution (0.9% w/v NaCl). The strains were resuspended in 20 mL of the same solution and inoculated at a concentration of approximately 8.2–8.5 log cfu/ml in phosphate/citrate buffer (obtained by mixing citric acid 0.3 M and Na_2_HPO_4_ 0.6 M solutions) added with tyrosine 4.4 mM and incubated at 37°C for 48 h. The determination of the effect of pH on TDC activity was performed in the phosphate/citrate buffer at pH values of 7, 6, 5, and 4 and incubated at 37°C. The effect of NaCl was determined at 37°C in buffer at pH 5 adding 0, 5, 10, and 15% (w/v) of NaCl while the influence of temperature was monitored by incubation at 20, 30, 37, and 45°C in buffer at pH 5 and with no NaCl added. At defined times (0, 2, 8, 24, and 48 h) the cell viability was assessed by plate counting in BHI Agar (Oxoid, Basingstoke, UK) incubated for 48 h at 37°C. In addition, the number of enterococci was determined after 48 h with Burker counting chamber to assess the proportion of undamaged (not lysed) cells. After 2 and 24 h of incubation, tyramine accumulation was determined.

### Purified TDC Enzyme Activity

At the same conditions described above, also the activity of a purified TDC (Sigma–Aldrich, Gallarate, Italy) was monitored. The pure enzyme, obtained from *E. faecalis* according to the producer, was added at 0.15 U/100 mL of phosphate/citrate buffer in the different conditions. At defined times (2, 4, 8, 24, and 48 h) the tyramine accumulation was assessed by the HPLC method described below.

### Biogenic Amine Determination

One ml of each culture obtained according to the condition described in paragraph 2.1 was centrifuged at 10000 rpm for 10 min at 10°C; pellet and supernatant were collected for further analysis. The supernatants were used for BAs determination by HPLC after derivatization with dansyl-chloride (Sigma–Aldrich, Gallarate, Italy) according to Tabanelli et al. (2012). The tyramine content was analyzed using a PU-2089 Intelligent HPLC quaternary pump, Intelligent UV–VIS multiwavelength detector UV 2070 Plus (Jasco Corporation, Tokio, Japan) and a manual Rheodyne injector equipped with a 20 μl loop (Rheodyne, Rohnert Park, CA, USA). The quantification of tyramine was performed as follows: gradient elution 0–5 min phosphate buffer (pH 7.0)/acetonitrile 35:65, 5–6 min water/acetonitrile 20/80, 6–15 min water/acetonitrile 10/90, 15–25 min phosphate buffer (pH 7.0)/acetonitrile 35:65 with flow rate 0.8 mL/min. The amount of tyramine was expressed as mM by reference to a calibration curve obtained with standard solutions.

### Analysis of the TDC Operon Region

Total genomic DNA was extracted from cell pellets using the Wizard Genomic DNA purification system (Promega Corporation, Madison, WI, USA), according to the manufacturer’s instructions. The TDC operon fragments were obtained for each strain by PCR amplification with the partially degenerate primers reported in **Table 1**. PCR mixture was composed of 1 × PCR buffer, 1.5 mM MgCl2, 200 nM dNTPs, 0,5 μM each primer, and 50 ng DNA. Amplification program comprised: 95°C for 5 min, 35 cycles at 94°C, 30 s; 56°C, 45 s; 72°C, 1 min and final extension at 72°C, 10 min. Amplicons were purified with the Wizard SV gel and PCR clean-up system (Promega, Italy), and cloned with the cloning kit pGEMT-easy vector system (Promega, Madison, WI, USA). Recombinant plasmids were sequenced at the GATC Biotech Ltd (Koln, Germany).

**Table 1:** Newly designed primers used in this study and expected size of the amplicons.

		Amplicon (pb)	
Primer code	Sequence (5′–3′)	*Enterococcus faecalis*	*Enterococcus faecium*
TyrS-F1	GGA GCT ATA AGT ATT AAC GGT GA	957	943
Tdc-R1	GAT TT(A/G) ATG TT(A/G) CG(G/C) GCA TAC CA		
Tdc-F2	CAA ATG GAA GAA GAA GT(A/T) GGA	1287	1340
Tdc-R2	CC(A/G/T) GCA CG(G/T) T(C/T)C CAT TCT TC		
Tdc-F3	CCA GA(C/T) TAT GGC AA(C/T) AGC CCA	819	784
TyrP-R3	CCT AAA GTA GAA GC(A/G) ACC AT		
TyrP-F4	TGG GTG CAA ATG TTC CCA GG	839	940
TyrP-R4	ACC (A/G)AT TCG (A/G)TA AGG ACG		
TyrP-F5	(A/T)CT GCT TGG GT(A/T) ACT GGA CC	1098	1056
NhaC-R5	CAT (C/T)GC AT(C/T) (A/G)T(C/T) GAA TCC AAG		
NhaC-F6	GTG TCT TAG TTG CT(A/T) C(A/T)T GGA T	1017	1017
NhaC-R6	CCA TAA TGA A(G/T)G T(A/G)C C(A/G)C T(A/G)A CT		

Promoters prediction was carried out by BPROM, a bacterial sigma70 promoter recognition program ^1^ (Solovyev and Salamov, 2011). Putative Rho–independent transcription terminators were predicted by the Arnold Finding Terminators^2^. The search procedure uses two complementary programs, Erpin (Gautheret and Lambert, 2001) and RNAmotif (Macke et al., 2001).

Similarity searches were performed with the Basic Local Alignment Search Tool (BLAST) available at the National Center for Biotechnology Information (NCBI^3^). Sequence alignments were carried out with the ClustalW2 analysis Tool Web Services from the EMBL-EBI (McWilliam et al., 2013).

### Statistical Analysis

Biogenic amine values and enterococci counts for each strains and for each condition are the mean of three different samples. The presence of significant differences was tested with ANOVA, using the Tukey HSD test carried out with Statistica 6.1 (StatSoft Italy srl, Vigonza, Italy).

## Results and Discussion

### Purified TDC Enzyme Activity

The amount of tyramine produced in phosphate/citrate buffer containing the purified TDC (0.15 U/100 ml) under different conditions is reported in **Table 2**. Similar amounts of tyramine (ranging from 0.60 to 0.59 mM) were detected at pH 5 and 6 after 24 h of incubation, suggesting that the optimum pH for TDC activity was comprised between these values. At pH 7, the amine production was drastically reduced (0.26 mM), while at pH 4 it was negligible (less than 0.02 mM). Shorter or longer incubation periods decreased and increased, respectively, the tyramine detected without significantly changing the proportion of BA produced.

**Table 2:** Tyramine produced in phosphate/citrate buffer containing the purified commercial TDC (0.15 U/100 ml) under different conditions.

	Tyramine (mmol/l)											
	37°C, 0% NaCl				pH 5, 37°C				pH 5, 0% NaCl			
Hours	pH 4	pH 5	pH 6	pH7	0% NaCl	5% NaCl	10% NaCl	15% NaCl	20°C	30°C	37°C	45°C
0	-	-	-	-	-	-	-	-	-	-	-	-
2	0.02 (±0.01)	0.14 (±0.02)	0.14 (±0.01)	0.08 (±0.01)	0.14 (±0.01)	0.14 (±0.00)	0.11 (±0.01)	0.10 (±0.01)	0.11 (±0.00)	0.14 (±0.01)	0.14 (±0.01)	0.13 (±0.00)
4	0.03 (±0.01)	0.19 (±0.03)	0.19 (±0.01)	0.11 (±0.02)	0.19 (±0.02)	0.18 (±0.01)	0.16 (±0.01)	0.13 (±0.00)	0.15 (±0.01)	0.19 (±0.02)	0.19 (±0.01)	0.18 (±0.01)
8	0.04 (±0.01)	0.28 (±0.03)	0.28 (±0.02)	0.16 (±0.03)	0.28 (±0.01)	0.24 (±0.02)	0.21 (±0.02)	0.18 (±0.01)	0.20 (±0.01)	0.27 (±0.02)	0.28 (±0.02)	0.27 (±0.01)
24	0.02 (±0.01)	0.60 (±0.04)	0.59 (±0.04)	0.26 (±0.02)	0.60 (±0.02)	0.50 (±0.03)	0.42 (±0.02)	0.33 (±0.02)	0.39 (±0.01)	0.55 (± 0.04)	0.60 (±0.03)	0.43 (±0.02)
48	0.03 (±0.01)	0.88 (±0.06)	0.95 (±0.06)	0.37 (±0.03)	0.88 (±0.03)	0.81 (±0.02)	0.70 (±0.04)	0.54 (±0.03)	0.56 (±0.02)	0.92 (±0.05)	0.88 (±0.04)	0.64 (±0.03)

*Standard deviation is reported within brackets.*

The increase of NaCl concentration determined a progressive and constant decrease of the efficacy of the enzymatic activity, throughout all the incubation period. However, the purified TDC maintained a high effectiveness even at 15% NaCl (about 62% of the tyramine produced in the absence of salt).

Regarding the effect of temperature, tyramine was produced in higher amount, and with higher rate, at 30 and 37°C (without significant differences between these two temperatures) and, after 24 h incubation, 0.55 and 0.60 mM of tyramine were detected, respectively. Slower decarboxylation kinetics were observed at 45 and 20°C. However, the final amount of tyramine was rather high, about the 65% of those observed under optimal conditions, indicating a good enzymatic activity also at the minimum and maximum temperature considered in these trials.

Liu et al. (2014) studied the effect of pH, temperature, and salt concentration on TDC activity from two strains of *E. faecalis* and *E. faecium* and heterologously expressed in *E. coli*. They found an optimum pH for tyrosine decarboxylation at 5.5 for the enzyme from *E. faecalis* and 6.0 for the enzyme from *E. faecium*. By contrast, the optimum temperature coincided for the two enzymes and was lower (25°C) than that found here. The same authors observed no effect on TDC activity of NaCl concentration up to 4.5%. Moreno-Arribas and Lonvaud-Funel (1999) demonstrated a maximum tyrosine decarboxylase activity at pH 5.5 in cell free extracts of two *L. brevis* strains. In addition, the TDC activity of cell free extract was always higher if compared with the activity of whole cells in relation not only to pH, but also to citrate, lactate, ethanol and tyramine concentration in the medium.

### Cell Viability

The viability of the cells was checked at different times (2, 8, and 24 h incubation) and **Table 3** reports the diminution of the log cfu/ml with respect to the initial inoculum (approximately 8.2–8.5 log cfu/ml).

**Table 3:** Viability loss of the strains inoculated in the different buffers after 2, 8, and 24 h of incubation, expressed as diminution of the log cfu/ml with respect to the initial inoculum.

	Cell load reduction											
	37°C, 0% NaCl				pH 5, 37°C				pH 5, 0% NaCl			
Hours	pH 4	pH 5	pH 6	pH 7	0% NaCl	5% NaCl	10% NaCl	15% NaCl	20°C	30°C	37°C	45°C
**EF37**											
2	0.13	0.04	0.18	0.11	0.04	0.01	0.16	0.13	0.16	0.03	0.04	0.03
8	0.26	0.03	0.32	0.17	0.03	0.14	0.14	0.25	0.16	0.10	0.03	0.09
24	1.64	0.09	0.65	0.60	0.09	0.18	0.17	0.43	0.17	0.12	0.09	0.01
**ATCC 29212**											
2	0.11	0.06	0.19	0.21	0.06	0.17	0.06	0.28	0.06	0.11	0.06	0.03
8	1.12	0.07	0.31	0.34	0.07	0.28	0.41	0.31	0.32	0.46	0.07	0.43
24	3.45	0.29	0.65	0.59	0.29	0.48	0.29	1.37	0.29	0.65	0.29	0.66
**FC12**											
2	0.04	0.03	0.07	0.14	0.03	0.03	0.11	0.03	0.11	0.05	0.03	0.01
8	0.81	0.09	0.47	0.62	0.09	0.16	0.12	0.02	0.32	0.53	0.09	0.46
24	2.57	0.30	0.57	0.69	0.30	0.51	0.24	1.57	0.14	0.52	0.30	0.43
**FC643**											
2	0.09	0.01	0.01	0.08	0.01	0.11	0.13	0.25	0.34	0.13	0.01	0.16
8	0.31	0.13	0.28	0.35	0.13	0.21	0.08	0.34	0.27	0.33	0.13	0.54
24	5.29	0.31	0.66	0.58	0.31	0.77	0.64	0.60	0.20	0.20	0.31	2.17

*Enterococcus faecalis* EF37 was characterized by a reduced loss of viability after 2 and 8 h, while after 24 h a marked reduction was observed at pH 4 (1.64 log cfu/ml). In all the other cases, the viability loss was always below 0.65 log unit and the strain showed the higher level of survivors in the presence of salt if compared with the other strains.

Also *E. faecalis* ATCC 29212 presented a reduced diminution in viable counts after 2 h (less than 0.30 log cfu/ml). After 8 h, the diminution was always lower of 0.5 log unit, with the exception of pH 4 at which the decrease was higher than 1 log unit. After 24 h of incubation high viability losses were observed at pH 4 (3.45 log cfu/ml) and in the presence of 15% NaCl (1.37 log cfu/ml).

An analogous behavior was shown by *E. faecium* FC12, with the higher level of cell death found after 24 h at pH 4 (2.57 log cfu/ml) and at 15% NaCl (1.57 log cfu/ml).

Finally, also *E. faecium* FC643 was characterized by a dramatic loss of viability after 24 h especially at 45°C (2.17 log cfu/ml) and at pH 4 (5.29 log cfu/ml).

Independently on the results of plate counting, after 24 h, the number of whole enterococcal cells was evaluated with a Burker chamber. No significant differences were found with the initial inoculum (data not shown). Thus, this suggests that, at least within 24 h, the enterococcal loss of viability was not associated to cell lysis, with a consequent release of cell decarboxylase in the buffer.

### Tyramine Production

**FIGURE 1:**
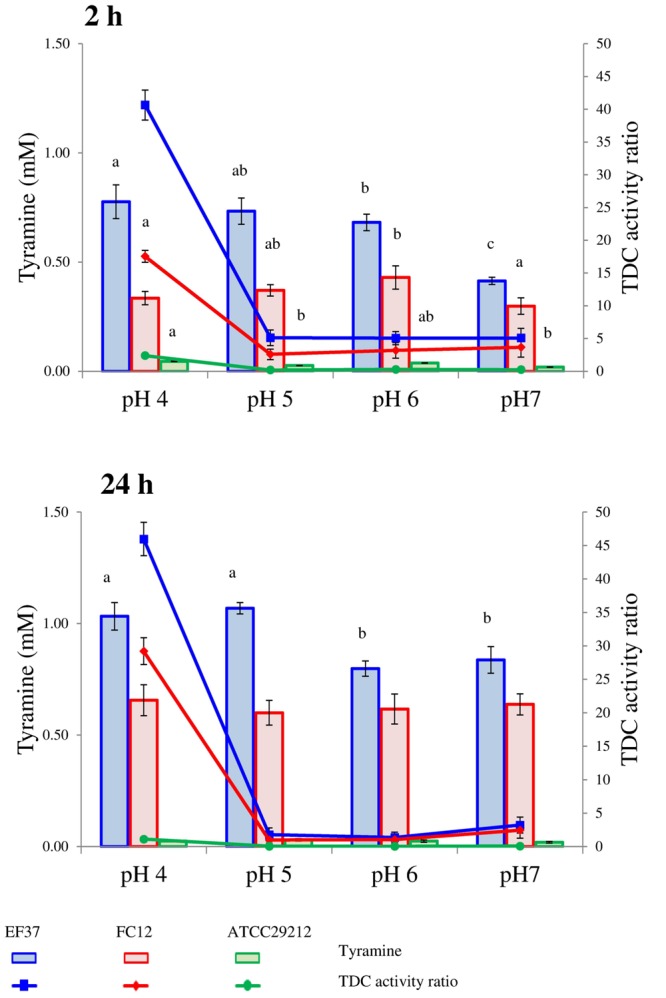
**Tyramine produced by enterococcal strains in phosphate/citrate buffer having different pH values after 2 and 24 h of incubation at 37°C.** When ANOVA was significant (*P* ≤ 0.05) lower-case letters are reported. For the same strain, values with the same letter are not statistically different (*P* > 0.05) according to the *post hoc* comparisons of the ANOVA. In the same graphs also the TDC activity ratio is shown, i.e., the ratio of mM of tyramine accumulated by cells and mM of tyramine produced by commercial TDC pure enzyme (0.15 U/100 ml) in the same conditions.

**FIGURE 2:**
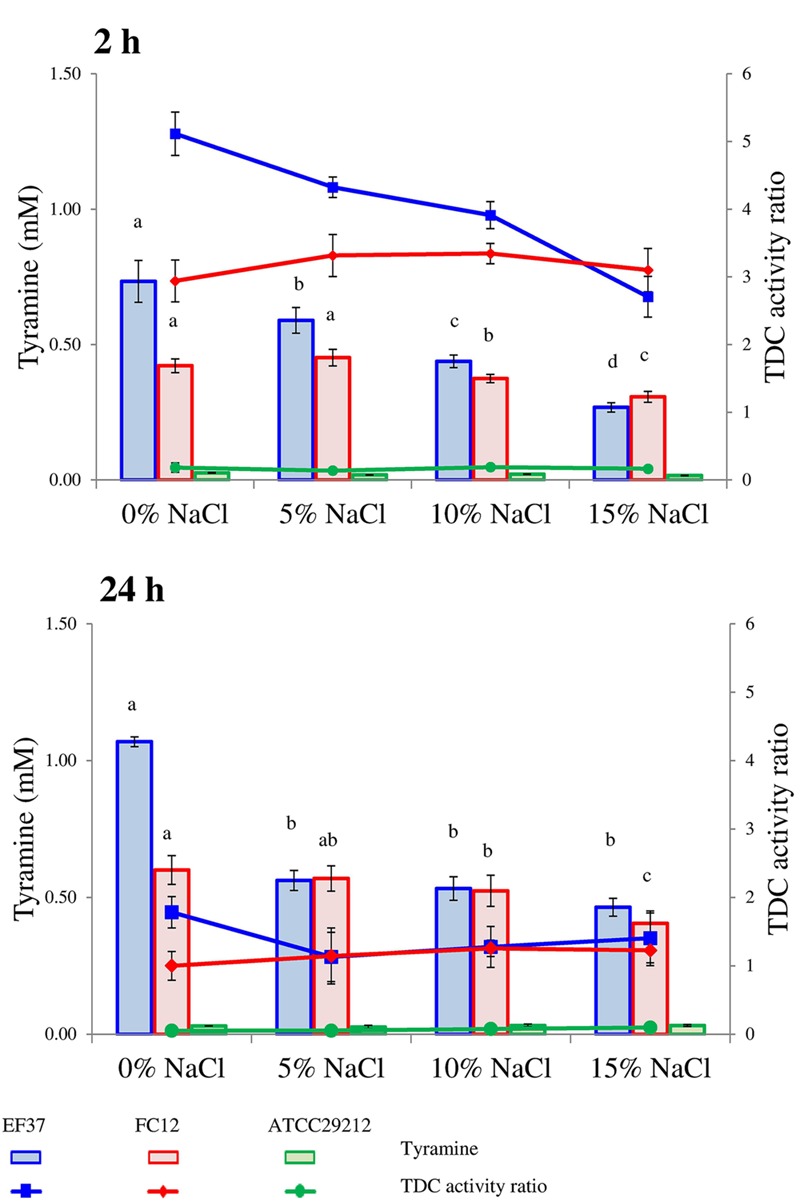
**Tyramine produced by enterococcal strains in phosphate/citrate buffer added with different amounts of NaCl after 2 and 24 h of incubation at 37°C.** When ANOVA was significant (*P* ≤ 0.05) lower-case letters are reported. For the same strain, values with the same letter are not statistically different (*P* > 0.05) according to the *post hoc* comparisons of the ANOVA. In the same graphs also the TDC activity ratio is shown, i.e., the ratio of mM of tyramine accumulated by cells and mM of tyramine produced by commercial TDC pure enzyme (0.15 U/100 ml) in the same conditions.

**FIGURE 3:**
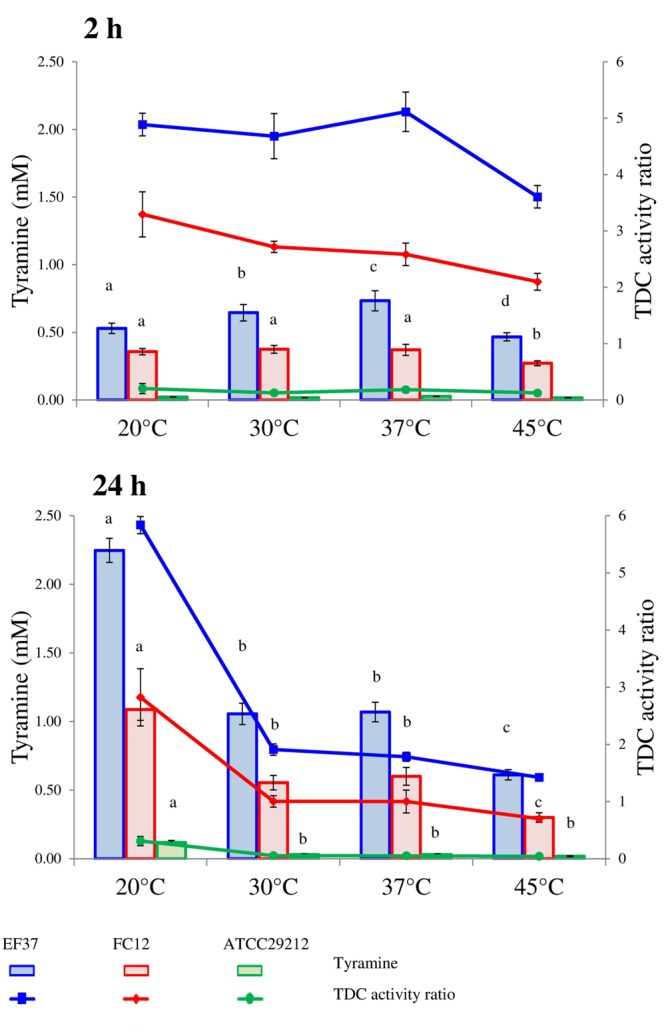
**Tyramine produced by enterococcal strains in phosphate/citrate buffer incubated at different temperatures after 2 and 24 h of incubation at 37°C.** When ANOVA was significant (*P* ≤ 0.05) lower-case letters are reported. For the same strain, values with the same letter are not statistically different (*P* > 0.05) according to the *post hoc* comparisons of the ANOVA. In the same graphs also the TDC activity ratio is shown, i.e., the ratio of mM of tyramine accumulated by cells and mM of tyramine produced by commercial TDC pure enzyme (0.15 U/100 ml) in the same conditions.

Tyramine concentrations detected after 2 and 24 h for the tested strains in relation to pH, NaCl concentration and incubation temperatures are reported in **Figures 1–3**, respectively. All the strains were pre-cultured in presence of the precursor to activate the transcription of the TDC gene cluster (Bargossi et al., 2015). The pre-adaptation in media containing tyrosine also allowed a rapid beginning of decarboxylase activity, with a detectable tyramine concentration since 2 h of incubation.

Under the adopted conditions, the strains EF37, ATCC 29212, and FC12 were able to decarboxylate amounts of the tyrosine supplied far from the maximum theoretical yield (4.4 mM). In particular, EF37 and FC12 produced the higher tyramine concentrations, while ATCC 29212 showed a reduced decarboxylase activity in all the conditions assayed (**Figures 1–3**). By contrast, *E. faecium* EF643 was not able to accumulate detectable amounts of tyramine in any of the tested conditions in 24 h and, for this reason, it is not present in the Figures. These different aptitudes of the tested strains confirm the trends of tyramine accumulation during growth in more complex systems, i.e., BHI and Bover-Cid and Holzapfel medium (Bargossi et al., 2015). In fact, in these media *E. faecalis* EF37 and *E. faecium* FC12 have shown an early tyramine accumulation (since the exponential phase) and the maximum BA concentration was reached at the beginning of the stationary phase. By contrast, the accumulation of tyramine by *E. faecalis* ATCC 29212 and *E. faecium* FC643 has been delayed and characterized by a slower rate while the amine has been accumulated in relevant amounts only during the stationary phase.

The strain *E. faecalis* EF37 generally showed the highest ability to decarboxylate tyrosine in citrate/phosphate buffer while *E. faecium* FC12 accumulated slightly lower amounts of tyramine. On the other hand, *E. faecalis* ATCC 29212 showed under all the conditions tested an extremely low aptitude to produce this BA. In fact, in all the samples, both after 2 and 24 h of incubation, the amount was always below 0.15 mM.

Regarding pH (**Figure 1**), *E. faecalis* EF37 accumulated higher amounts of tyramine at the lower pH values, with more marked differences after 24 h. Tyramine production of *E. faecium* FC12 was weakly affected by pH, and the amounts detected varied between 0.34 and 0.43 mM after 2 h and 0.60 and 0.66 mM after 24 h of incubation.

The tyrosine decarboxylation pathway is reported to contribute to an acid response mechanism in *E. faecium* because it gives to the strain a competitive advantage in nutrient-depleted conditions, as well as in harsh acidic environments (Pereira et al., 2009). The same role in the maintenance of pH homeostasis in acidic environment has been described also in *E. durans* (Linares et al., 2009), *E. faecium* (Marcobal et al., 2006), and *E. faecalis* (Perez et al., 2015). These latter authors have reported that tyrosine decarboxylation pathway improves survival under acidic conditions. This could explain the higher cell viability loss at pH 4 of *E. faecium* FC643, which did not produce tyramine, and of *E. faecalis* ATCC 29212, which accumulated low amounts of amine. However, in this perspective, the behavior of *E. faecium* FC12, which did not present significant differences in relation to pH, appears to be surprising and needs further investigations. On the other hand, the purified TDC showed a low activity at pH 4 (**Table 2**) and the environmental pH influenced the overall cell metabolism rather than the specific activity of TDC inside the cytoplasm. In other words, the heterogeneity found in the enterococcal strains could be related to the general physiological state of the cells which, in turn, influenced the TDC activity.

**Figure 2** reports the tyramine accumulation in relation to NaCl concentrations. The addition of increasing amounts of salt caused a progressive diminution in tyrosine decarboxylation by *E. faecalis* EF37 after 2 h. This strain accumulated 0.73 mM of tyramine in the absence of NaCl and tyramine decreased to 0.59 mM in the presence of 5% NaCl and 0.27 mM with 15% NaCl. After 24 h, small differences in tyramine content (ranging from 0.46 to 0.57 mM) were observed in the presence of the different NaCl concentrations, while this amount was about doubled in the control sample without salt (1.07 mM). The decarboxylating activity of FC12 was not particularly affected by increasing NaCl concentrations, except for a partial reduction at 15% NaCl, both after 2 and 24 h of incubation. The tyramine production by *E. faecalis* ATCC 29212 was negligible, without differences in relation to salt modulation.

A general reduction of tyramine accumulation by enterococci due to NaCl concentration has already been described both *in vitro* (Liu et al., 2014 and in fermented foods (Gardini et al., 2008). In addition, the same trend was observed using the purified TDC, as evidenced in **Table 2**.

Buňková et al. (2011) showed that the presence of the higher NaCl concentration tested (2%) favored the accumulation of tyramine by *Lactococcus lactis* strains that started during the active growth phase of the cells.

For *E. faecalis* EF37, the effect of the temperature was noteworthy. In fact, after 2 h, the higher tyramine accumulation was observed at 37°C (0.73 mM), which is the optimal temperature for enterococci, while for the purified enzyme no differences were observed within 30 and 37°C. Lower tyramine concentrations were found at 20 and 45°C (0.53 and 0.47 mM, respectively). By contrast, after 24 h of incubation, the highest content of tyramine were found at 20°C (2.25 mM), while in the samples incubated at 30 and 37°C this concentration was halved. In the trial at 45°C the concentration remained quite stable (0.60 mM) with respect to sample collected after 2 h.

For *E. faecium* FC12, after 2 h of incubation, no significant differences were found between 20 and 37°C, while the BA concentration was reduced at 45°C. This lower production at the higher temperature tested was found also after 24 h of incubation. In addition, at the lowest temperature (20°C) the highest tyramine accumulation was observed as already found for *E. faecalis* EF37. This behavior was confirmed also for *E. faecalis* ATCC 29212, which accumulated the highest amount of amine in the sample incubated at 20°C for 24 h (0.12 mM). In general, the effect of temperature has been tested in relation to the growth of the cells and, under these conditions, lower incubation temperature are associated with lower BA accumulation (Masson et al., 1996; Gardini et al., 2001). Under the conditions applied to the cells suspended in the buffer, the higher tyramine levels were detected at the lower temperature, suggesting the possibility that the tyrosine decarboxylase activity can be highly activated under not favorable temperature.

The lines drawn in **Figures 1–3** represent the ratio between the tyramine produced by the cells in the buffer and the tyramine accumulated under the same conditions by the purified enzyme (reported in **Table 2**). These values were added with the aim to highlight the different performances of TDC when it worked inside or outside the cells.

First of all, this index reflects the inability of *E. faecalis* ATCC 29212 to decarboxylate tyrosine under the adopted conditions. In the other two strains, the decarboxylase activity after 2 h of incubation is always higher than in samples with the purified enzyme and, consequently, the ratio was higher than 1. By contrast, after 24 h, the amine produced by the purified enzyme and the enterococcal strains was comparable with ratio close to 1. A noteworthy exception to this trend was represented by the samples inoculated with *E. faecalis* EF37 and *E. faecium* FC12 incubated at 20°C and at pH 4. In these cases, the decarboxylase activity was extremely higher in the presence of cells and the ratios were comprised between 3 and 6 at 20°C and 30 and 45 at pH 4.

In other words, the decarboxylating activity inside the cells is influenced by the chemico-physical factors through two different mechanisms; on one side the environmental factors directly affect the enzyme activity while, on the other side, they regulate the overall cell metabolism and, in turn, the rate of exchange between inside and outside. The interaction between these two aspects can explain the different responses of tyramine accumulation observed using the purified enzyme and the viable cells. In this perspective, it is noteworthy the cell response at 20°C of the strains EF37 and FC12 in which the production of tyramine seems to be an important strategy for the enterococcal growth at this unfavorable temperature.

Therefore, the final results of the decarboxylase activity, i.e., the amount of BA accumulated, depends on the activity of TDC which works inside the cell, but also by the ability of the cells to transport the precursor in the cytoplasm and to excrete the final product (tyramine) outside. This antiport, driven by the tyrP activity (Marcobal et al., 2012), could be affected by the environmental conditions.

### Analysis of the TDC Operon Region

**FIGURE 4:**
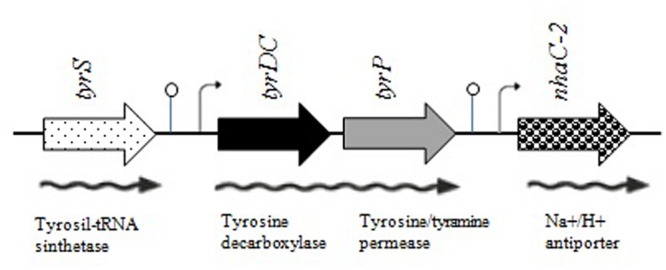
**Genetic organization of the TDC region containing the genes *tyrS, tyrDC, tyrP*, and *nhaC-2* in the enterococcal strains studied.** ORFs are represented by arrows, putative promoters by broken arrows, transcription terminator regions by lollipops. Expected mRNA and enzymatic function are also indicated (adapted from Marcobal et al., 2012).

To characterize the TDC pathway of the four enterococcal strains, we amplified and sequenced the region carrying the genes *tyrDC, tyrP*, and *nhaC-2*. The six sets of newly designed primers amplified overlapping fragments of the expected size (**Table 1**) for all the strains including *E. faecalis* ATCC 29212 which was used as a control, since its complete genome sequence was already available (gb|CP008816.1|; Minogue et al., 2014). The TDC cluster sequence of all the strains shares the same genetic organization, which comprises, downstream the gene *tyrS*, the three predictable complete open reading frames (ORF) corresponding to the genes *tyrDC, tyrP*, and *nhaC-2*. They are oriented in the same direction and encode polypeptides larger than 300 amino acids (**Figure 4**).

Sequence analysis performed by the software BPROM and Arnold did not revealed the presence of putative promoters and terminators between the genes *tyrDC* and *tyrP*, indicating that they could be co-transcribed. Putative promoters and terminators were conversely found upstream the start codon of the genes *tyrDC* and *nhaC-2* (**Figure 4**). This suggests that the expression of *tyrDC* and *tyrP* is probably independent from that of the flanking genes *tyrS* and *nhaC-2*. Different polycistronic mRNA transcripts have been described in enterococcal strains, such as *tyrS-tyrDC-tyrP* (Connil et al., 2002), *tyrDC-tyrP* (Linares et al., 2009), *tyrDC-tyrP-nhaC-2*, and *tyrP-nhaC-2* (Perez et al., 2015). So *tyrDC* and *tyrP* can be transcribed from different manner and future Reverse-Transcription-PCR experiments are needed to clarify the transcriptional organization of the TDC gene cluster in the examined strains.

BLASTN analysis of the 5259 bp nucleotide sequence of *E. faecalis* EF37 TDC operon region showed an overall identity of 99% (5231/5259 bp) with that of *E. faecalis* ATCC 29212, and 100% identity (5259/5259 bp) with that of another completely sequenced strain of the same species, *E. faecalis* D32 (gb| CP003726.1|).

BLASTX analysis and comparison of the deduced amino acid sequences of the two *E. faecalis* strains was also carried out. These analyses revealed only two substitutions in amino acid sequences of EF37 and ATCC 29212. The first one (leucine to methionine) was found at the beginning of the ORF coding for the amino acid permease and the second one (isoleucine to valine) was located in the ORF corresponding to the Na^+^/H^+^ antiporter (position 18). These amino acid substitutions are conservative, and thus probably have no effect on the enzymatic activities.

Nucleotide sequence analysis of the *E. faecium* FC12 showed the highest identity (5293/5294 bp, 99%) with the operon of the strain *E. faecium* NRRL B-2354 (complete genome NC_020207), while the identity decrease to a value of 97% (5128/5296 bp) with the other *E. faecium* strain studied in this research.

As regards amino acid residues, BLASTX analysis of the TDC locus of FC12 showed a 100% of identity with TDCs (frame +3, 625 aa), amino acid permeases (frame +2, 456 aa) and Na+/H+ antiporters (frame +3, 414 aa) in the database. Amino acid sequences corresponding to the genes *tyrP* and *nhaC-2* of *E. faecium* FC643 were characterized by a 100% of identity with known amino acid permeases (frame +3, 456 aa) and Na+/H+ antiporters (391 aa) proteins, respectively. The TDC region translated sequence of FC643 showed an identity of 99% (624/625) with the amino acid sequences of almost all *E. faecium* strains present in database. However, a premature codon stop, introduced by a non-sense mutation (TGG/TAG) was found at position 40 of the protein. This mutation probably produces an abnormally shortened protein, supporting the inability of the strain FC643 to accumulate tyramine in the tested conditions at 24 h.

**FIGURE 5:**
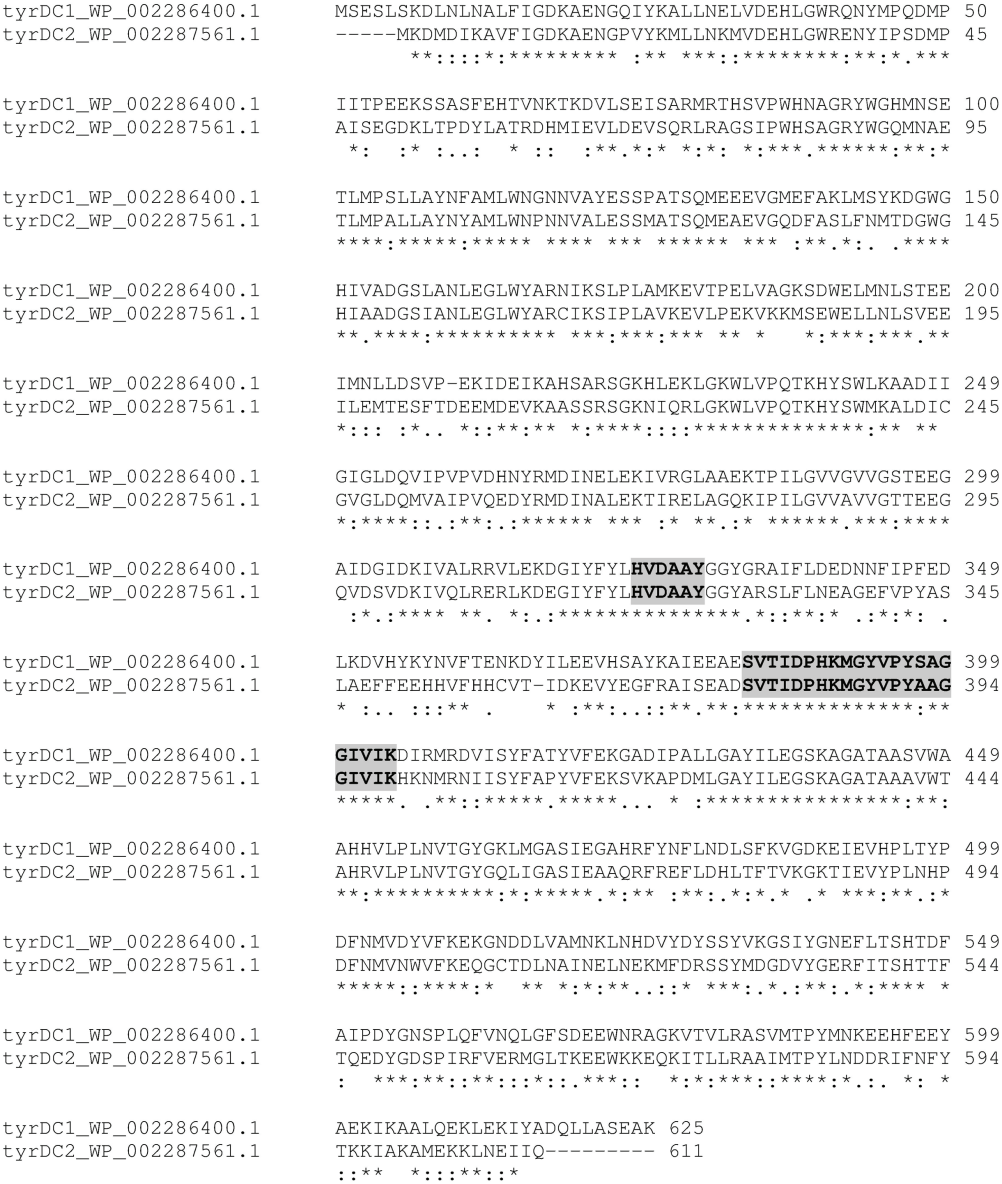
**Comparison of TDC protein sequences found in the genome of *Enterococcus faecium* Aus0085.** The HVDAAY conserved motif and PLP binding site characteristics of the group II PLP-dependent decarboxylase family are indicated in bold and highlighted in gray.

Nevertheless, Bargossi et al. (2015) highlighted the capacity of FC643 to accumulate lower level of tyramine in stationary phase of growth in complex media if compared with *E. faecium* FC12. Moreover, this strain did not accumulated 2-phenylethylamine in the same conditions. The decarboxylase activity of FC643, even if slow and reduced, in the presence of the premature codon stop in TDC region, could be ascribed to the presence of an additional gene coding for a decarboxylase enzyme involved in tyramine production. Indeed, comparison analysis of the *tyrDC* sequence to databanks allowed the identification of another gene coding for a putative tyrosine decarboxylase in all the publicly available whole genome sequences of *E. faecium*, i.e., *E. faecium* strains Aus0085 (AGS74230.1), NRRL B-2354 (AGE29157.1), DO (AFK57968.1), Aus0004 (AFC62424.1), and T110 (AII38451.1). This gene was not detected in the genome of *E. faecalis*. The additional enzyme has a nucleotide and amino acid identity score of 67–68% with the first known tyrosine decarboxylase, but it maintains catalytic residues involved in enzyme activity, the consensus pattern for pyridoxal phosphate-dependent decarboxylases where lysine (K) is the attachment site for the cofactor and the conserved LHVDAAY motif (Sandmeier, 1994; **Figure 5**). Nowadays no information are available about this additional enzyme that in the complete genomes of *E. faecium* is always followed by two amino acid permease coding genes. To elucidate the role of these proteins in *E. faecium* strains amino acid metabolism further investigations are needed.

## Conclusion

The tyramine production by cells re-suspended in buffered systems highlighted the heterogeneity of TDC activity within enterococci. The study of the genetic characteristics of the *E. faecium* strains allowed to correlate specific mutations in the *tyrDC* gene sequence to their different tyraminogenic activity, and suggested the involvement of another gene annotated as putative tyrosine decarboxylase in the complete genome of *E. faecium.* To our knowledge, the potential role of an additional decarboxylase enzyme with different substrate specificity and genetic organization was here noticed for the first time. The two *E. faecalis* strains showed highly conserved TDC operon region, thus their phenotypic behavior could be ascribed to different regulation mechanisms not yet elucidated, and affected by environmental factors or by the overall cell metabolism. In fact, the higher tyramine concentration produced by the enterococcal strains was found in the less favorable conditions for the purified TDC (at 20°C and pH 4). Further investigations have to be performed for better understanding the genetic determinants and mechanisms involved in tyramine production under different chemico-physical conditions by the application of “omic” approaches.

## Author Contributions

EB: perfomed the growth test in buffer system.

FG: elaborated the data and wrote part of the manuscript.

VG and ST: performed the genetic operon analysis.

CM: elaborated the data and wrote part of the manuscript.

GT: performed HPLC analysis and wrote part of the manuscript.

## Conflict of Interest Statement

The authors declare that the research was conducted in the absence of any commercial or financial relationships that could be construed as a potential conflict of interest.

## Footnotes

1 http://linux1.softberry.com/berry.phtml?topic=bprom&group=programs&subgroup=gfindb

2 http://rna.igmors.u-psud.fr/toolbox/arnold/index.php

3 http://www.ncbi.nlm.nih.gov
